# Supplementary material for: Aging-induced hepatocyte CD44 drives IL6/STAT3 signaling and associates with impaired neighboring T cell function
Source: bioRxiv. 2025 Dec 22:2025.12.20.695732. Preprint. [Version 1] doi: 10.64898/2025.12.20.695732 (PMC12776055; doi:10.64898/2025.12.20.695732)
Supplement: Supplement 2 [file NIHPP2025.12.20.695732v1-supplement-2.pdf]

bioRxiv preprint doi: https://doi.org/10.64898/2025.12.20.695732; this version posted December 22, 2025. The copyright holder for this preprint (which was not certified by peer review) is the author/funder, who has granted bioRxiv a license to display the preprint in perpetuity. It is made available under aCC-BY 4.0 International license.

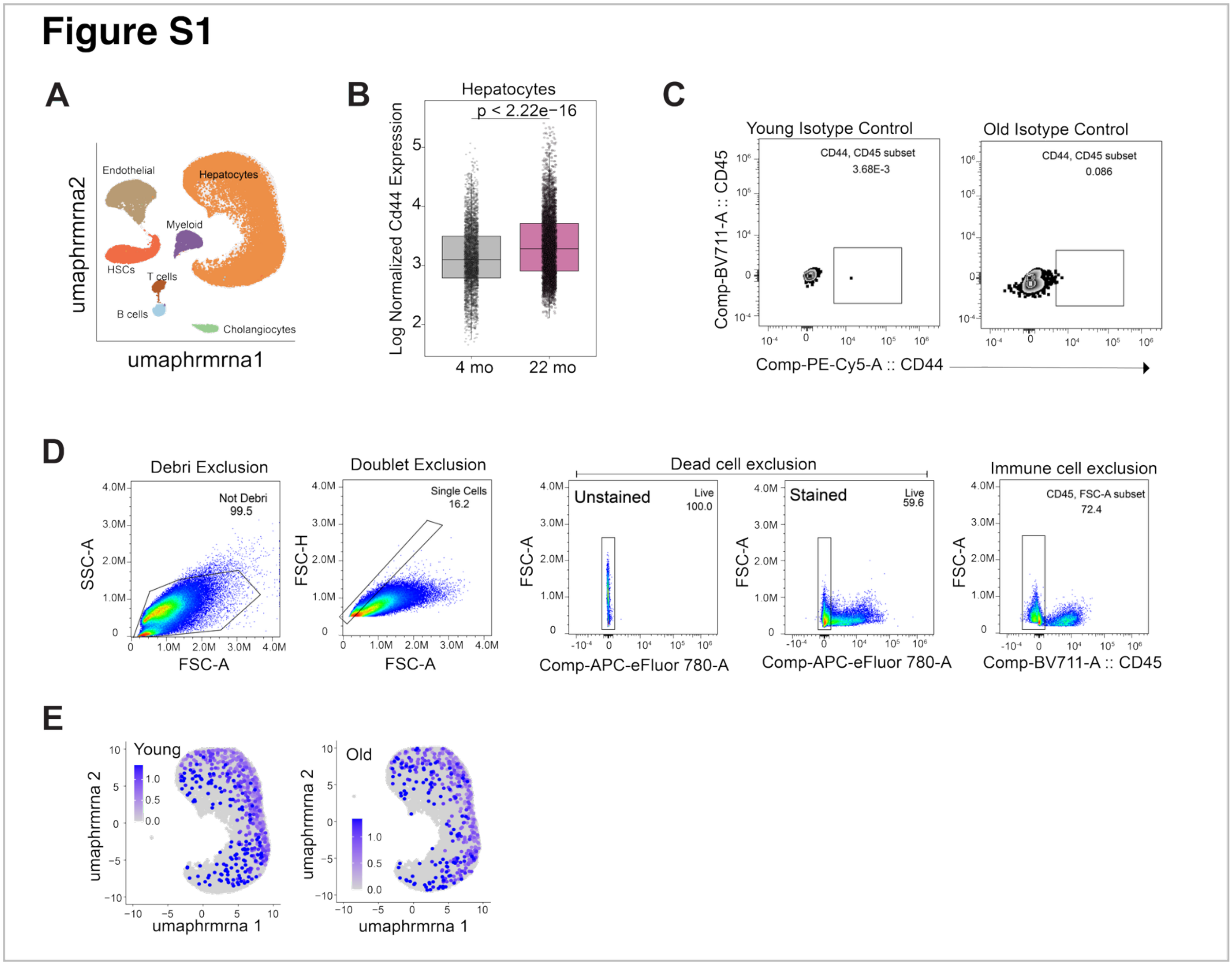

Figure S1. Abundance of CD44-expressing hepatocytes increases in aged mouse liver. A. Uniform Manifold Approximation and Projection (UMAP) of liver nuclei (10x multiome) from young and old C57BL/6J male mice, annotated by cell type (Endothelial, Hepatocytes, Myeloid, Hepatic stellate cells, T cells, B cells, Cholangiocytes). **B.** Box plot shows distribution of *Cd44* expression per hepatocyte with age. Y-axis represents log-normalized expression of *Cd44* in hepatocytes. **C.** Flow cytometry analysis of hepatocytes from young and old mouse livers. L-lobes were perfused, and hepatocytes were purified using Miltenyi perfusion kit. Flow plots show staining with isotype control antibodies in young and old isolated hepatocytes. **D.** Gating strategy for Fig. 1E and S1C. **E.** Single nucleus multiome profiling of young and old female mouse livers. UMAP shows *Cd44* transcript expression in hepatocyte nuclei from young (n = 3) and old (n = 3) female livers, each dot represents a nucleus.

bioRxiv preprint doi: https://doi.org/10.64898/2025.12.20.695732; this version posted December 22, 2025. The copyright holder for this preprint (which was not certified by peer review) is the author/funder, who has granted bioRxiv a license to display the preprint in perpetuity. It is made available under aCC-BY 4.0 International license.

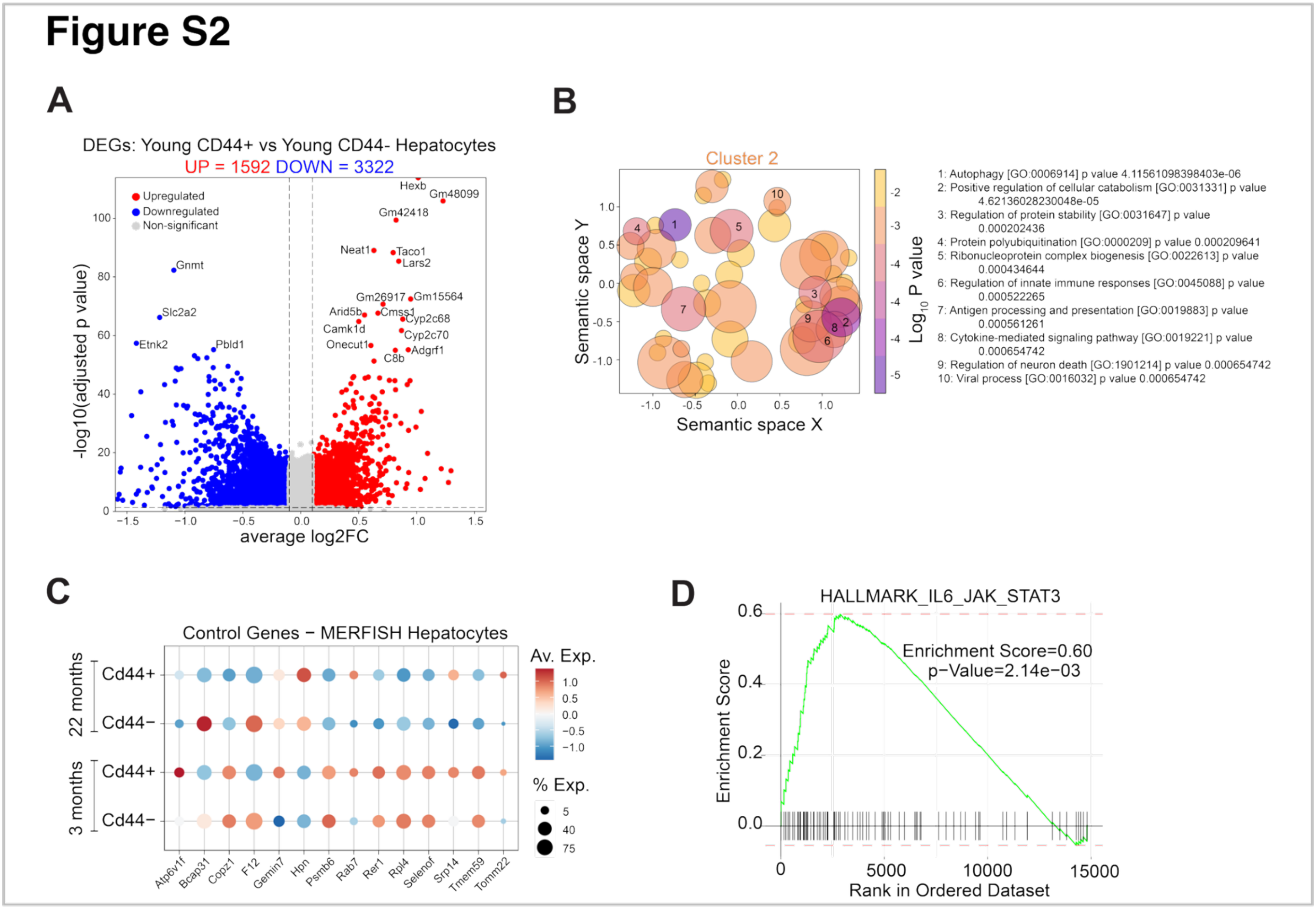

Figure S2. *Cd44* expressing hepatocytes in aged liver exhibit an immune modulatory transcriptome. A. Volcano plot depicting DEGs between *Cd44*+ versus *Cd44*- hepatocytes in young male livers. 1592 genes are upregulated and 3322 genes are downregulated in young *Cd44*+ hepatocytes compared to young *Cd44*- hepatocytes. (Log2FoldChange cutoff of 0.1 and -0.1 respectively, and adjusted p-value ≤ 0.05). **B.** GO term enrichment analysis of genes from Cluster 2 in Fig. 2D. GO terms are displayed in semantic space by GoFigure to reduce redundancy. Top 10 significant enriched terms labelled. **C.** Expression of control genes by MERSCOPE in hepatocytes defined as *Cd44*+ and *Cd44*- in young and old livers. Color indicates average expression and size of dot indicates percent of cells expressing cells. **D.** Gene Set Enrichment Analysis (GSEA) for IL-6/JAK/STAT3 signaling pathway in hepatocytes isolated from old livers compared to young livers[25]. Enrichment score = 0.6 in old hepatocytes compared to young hepatocytes, FDR = 2.14e–03.

bioRxiv preprint doi: https://doi.org/10.64898/2025.12.20.695732; this version posted December 22, 2025. The copyright holder for this preprint (which was not certified by peer review) is the author/funder, who has granted bioRxiv a license to display the preprint in perpetuity. It is made available under aCC-BY 4.0 International license.

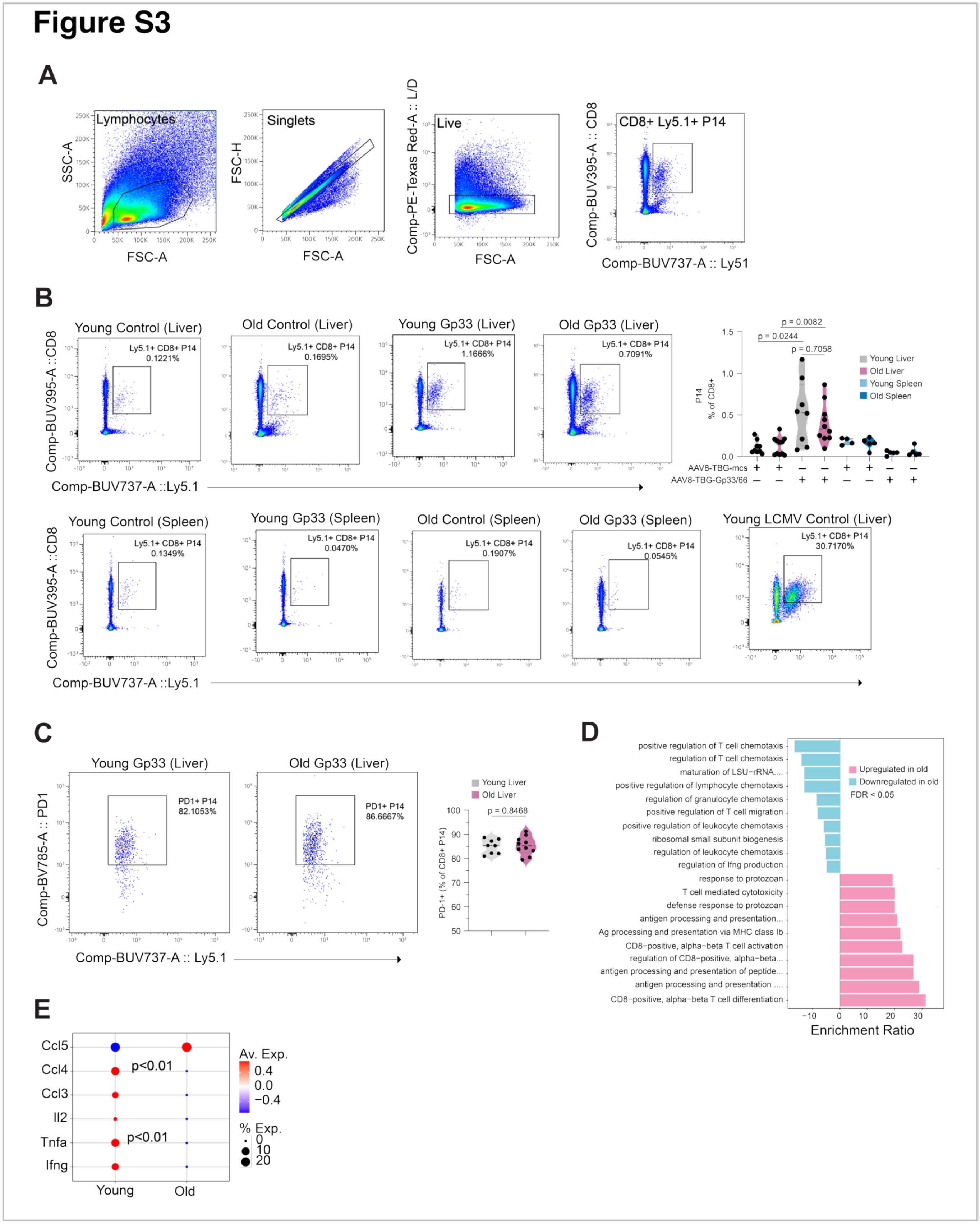

682

bioRxiv preprint doi: https://doi.org/10.64898/2025.12.20.695732; this version posted December 22, 2025. The copyright holder for this preprint (which was not certified by peer review) is the author/funder, who has granted bioRxiv a license to display the preprint in perpetuity. It is made available under aCC-BY 4.0 International license.

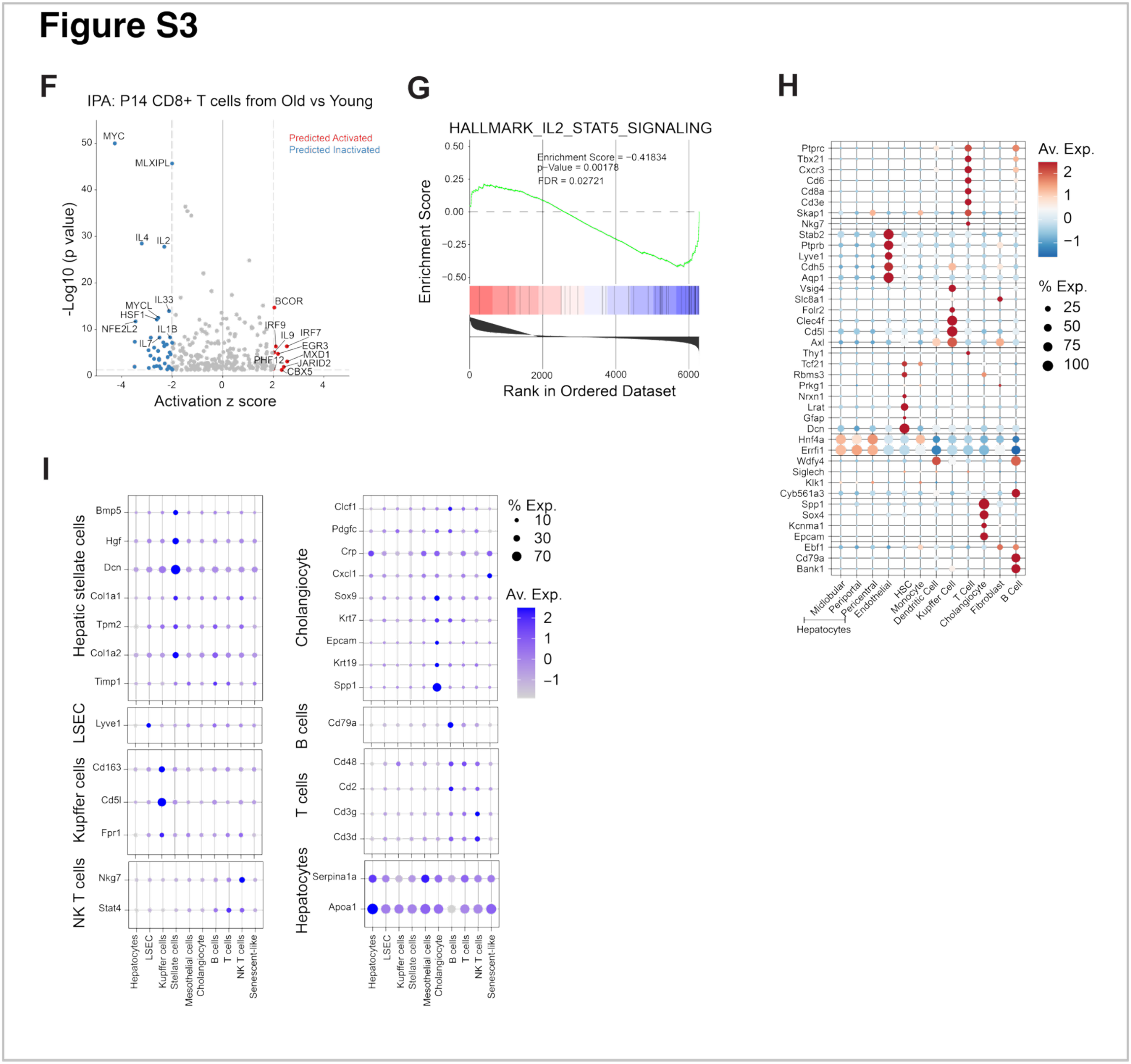

Figure S3. Aged livers harbor T cells with markers of inactivation. A. Gating strategy for analyzing Ly5.1+ P14 CD8+ T cells by flow cytometry. CD8+ Ly5.1+ P14 cells were gated on live (negative for PE-Texas Red) and single cells. Lymphocytes were first gated by forward and side scatter, followed by singlets (FSC-A vs. FSC-H) and live cells (PE–Texas Red–negative). From this live, single lymphocyte population, CD8^{+} Ly5.1^{+} P14 T cells were gated for analysis. **B.** Representative flow plots depicting staining for CD8 versus Ly5.1 in cell suspension of young and old livers and spleens of mice infected with AAV-TBG-mcs (control) and AAV-TBG-GP33 (antigen), and liver of young LCMV infected positive control. Violin plot quantifies the percentage of CD8+

bioRxiv preprint doi: https://doi.org/10.64898/2025.12.20.695732; this version posted December 22, 2025. The copyright holder for this preprint (which was not certified by peer review) is the author/funder, who has granted bioRxiv a license to display the preprint in perpetuity. It is made available under aCC-BY 4.0 International license.

Ly5.1+ cells in the total CD8+ T cell population recovered from young and aged, control or antigen infected livers; each dot represents one mouse (n = 9 young liver (control), n = 11 old liver (control), n = 8 young liver (antigen), n = 10 old liver (antigen), n = 4 young spleen (control), n = 5 old spleen (control), n = 5 young and old spleen (antigen). p values indicated for relevant comparisons in the plot. Data were analyzed using Kruskal–Wallis test and multiple comparisons were corrected using the two-stage linear step-up procedure of Benjamini, Krieger and Yekutieli. **C.** Representative flow plots show Ly5.1 versus PD-1 staining gated on CD8+ P14 population. Violin plot quantifies the PD-1+ as a percent of the CD8+ P14 population recovered from young and aged livers; each dot represents one mouse (n = 8 young, n = 10 old); p value was calculated by Unpaired t test (p = 0.8468, non-significant). **D.** GO term enrichment analysis of upregulated and downregulated DEGs (Significant (p < 0.05) with a log2FC of more than 1 or less than -1) from single cell RNA-seq of P14 CD8+ T cells from old recipient livers compared to young recipient livers. Cells pooled from n = 7 young and n = 7 old mouse livers. **E.** Dot plot showing expression of effector cytokines, *Il2* and chemokines from single cell RNA-seq of P14 CD8+ T cells recovered from young and old recipient livers. Color denotes average expression level and size of dot indicates percent of cells expressing the transcript. Significant p values indicated in the plot. Adjusted p values were calculated by Benjamni-Hochberg test. **F.** Ingenuity pathway analysis of DEGs comparing P14 CD8+ T cells recovered from young and old recipient livers shown in Fig. 3B. Volcano plot depicts top upstream predicted activated (Activation z-score ≥ 2) and predicted inactivated (Activation z-score ≤ –2) regulators in P14 CD8+ T cells recovered from old livers. **G.** GSEA for IL2/STAT5 signaling pathway in P14 CD8+ T cells recovered from old recipient livers compared to young livers. Enrichment score = –0.41834 in P14 cells recovered from old compared to young, FDR = 0.02721. **I.** and **J.** Cell type annotation marker expression plot for MERSCOPE and CosMx respectively.

bioRxiv preprint doi: https://doi.org/10.64898/2025.12.20.695732; this version posted December 22, 2025. The copyright holder for this preprint (which was not certified by peer review) is the author/funder, who has granted bioRxiv a license to display the preprint in perpetuity. It is made available under aCC-BY 4.0 International license.

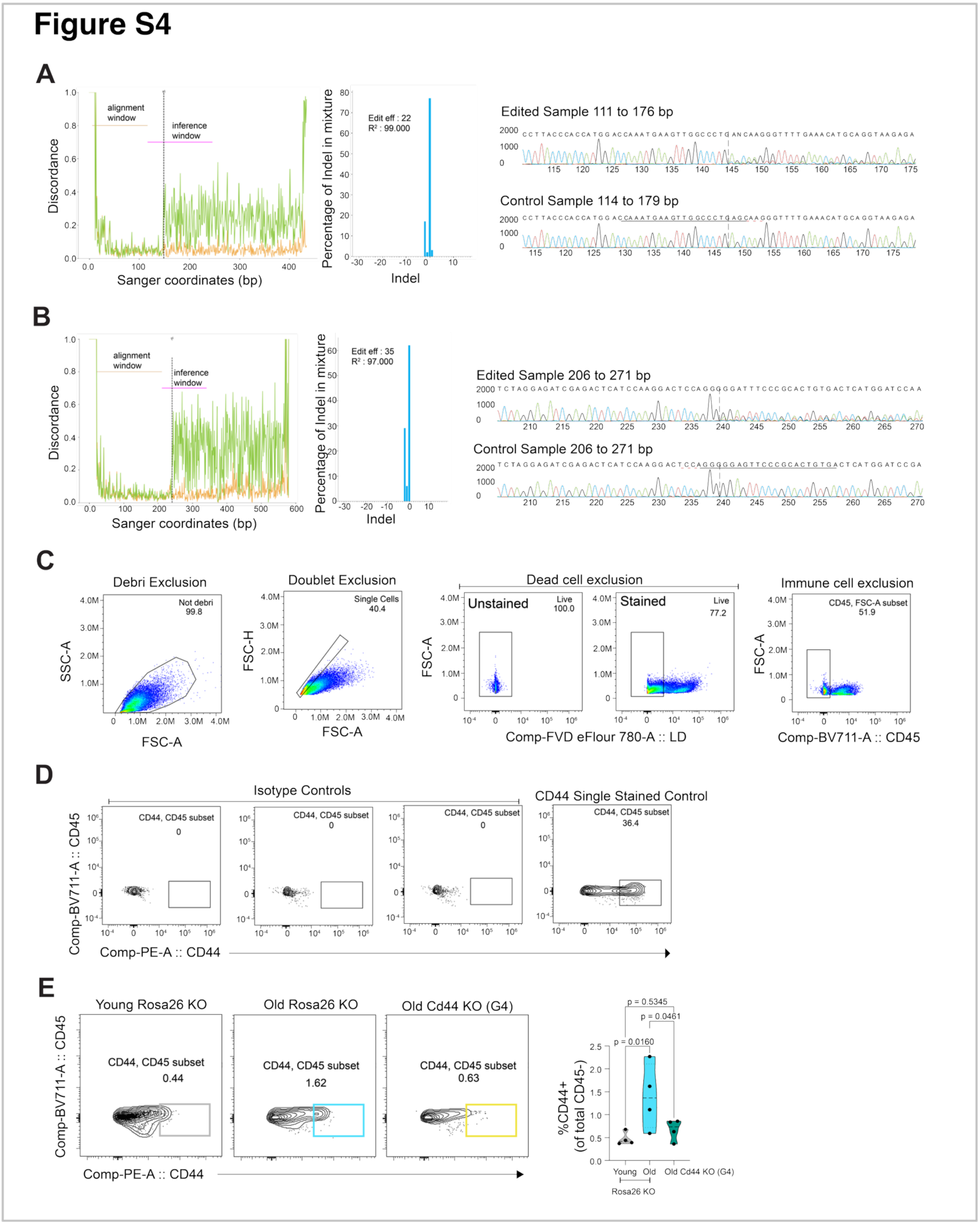

712

713 **Figure S4. Knock out of hepatocyte *Cd44* attenuates immune modulatory gene signatures in aged mouse**

bioRxiv preprint doi: https://doi.org/10.64898/2025.12.20.695732; this version posted December 22, 2025. The copyright holder for this preprint (which was not certified by peer review) is the author/funder, who has granted bioRxiv a license to display the preprint in perpetuity. It is made available under aCC-BY 4.0 International license.

livers. A. and **B.** Left: *Cd44* knockout in old livers using sgRNAg2 (A) and sgRNAg4 (B). Plot depicts discordance in the *Cd44* gene at the inference window. Middle: Insertions/deletions in *Cd44* gene are quantitated as Indel. Right: Corresponding edited sample sequence display with the control sample sequence. **C.** Flow cytometry analysis of CD44 in hepatocytes from young and old mouse livers. L-lobes were perfused, and hepatocytes were purified using Miltenyi perfusion kit. Flow plots show gating strategy for analyzing CD44 staining on hepatocytes. Hepatocytes were identified by first excluding debris and doublets, followed by gating on live cells and subsequently excluding CD45+ cells. **D.** Flow plots show staining with isotype controls of CD45- population from C for young and old isolated hepatocytes. CD44 single stained control was used for gating on CD44+ population. **E.** Representative flow plots show CD44 versus CD45 staining of CD45- cells from young Rosa26 control, old Rosa26 control and old Cd44 knockout with sgRNAg4. Corresponding violin plot quantifies the percentage of CD44+ population within the CD45 negative population by age; each dot represents one mouse (n = 4 young (Rosa26 control), n = 4 old (Rosa26 control), n = 4 old (Cd44 knockout). p values for all comparisons were determined by Ordinary one-way ANOVA with uncorrected Fischer's LSD in Prism 10.6.1.

Supplementary Data Table 1 (T1). Differentially expressed genes (DEGs) in young *Cd44*+ hepatocytes: full DEG list comparing young *Cd44*+ versus young *Cd44*– hepatocytes (depicted in Fig. S2A). DEGs were identified using the MAST method applied to single-nucleus RNA-seq data.

Supplementary Data Table 2 (T2). Differentially expressed genes (DEGs) in old *Cd44*+ hepatocytes: full DEG list comparing old *Cd44*+ versus old *Cd44*– hepatocytes (Fig. 2A). DEGs were identified using the MAST method applied to single-nucleus RNA-seq data.

Supplementary Data Table 3 (T3). Gene Ontology (GO) enrichment for genes uniquely upregulated in old Cd44+ hepatocytes: complete list of GO terms derived from enrichment analysis of the 186 genes uniquely upregulated in old Cd44+ hepatocytes.

bioRxiv preprint doi: https://doi.org/10.64898/2025.12.20.695732; this version posted December 22, 2025. The copyright holder for this preprint (which was not certified by peer review) is the author/funder, who has granted bioRxiv a license to display the preprint in perpetuity. It is made available under aCC-BY 4.0 International license.

Supplementary Data Table 4 (T4). Gene Ontology (GO) enrichment for Cluster 2 genes: complete list of GO terms derived from enrichment analysis of genes in Cluster 2 (depicted in Fig. 2D).

Supplementary Data Table 5 (T5). Upstream regulator analysis for Cd44+ hepatocyte DEGs in old liver: complete list of predicted upstream regulators identified from DEGs comparing *Cd44*+ versus *Cd44*– hepatocytes in old liver.

Supplementary Data Table 6 (T6). Single cell RNA seq of P14 CD8+ T cells recovered from old livers: full list of DEGs for P14 CD8+ T cells recovered from GP33-expressing old livers in comparison to young livers. Cells were pooled from n = 7 young and n = 7 old mouse livers.

Supplementary Data Table 7 (T7). Upstream regulator analysis for P14 CD8+ T cells recovered from old livers: complete list of predicted upstream regulators identified from DEGs in P14 CD8+ T cells recovered from old livers compared to young livers.
